# Influence of Circular through Hole in Pt–Rh Bushing on Temperature Propagation at High Temperature

**DOI:** 10.3390/ma15217832

**Published:** 2022-11-06

**Authors:** Nan Yang, Youping Gong, Peng He, Chuanping Zhou, Rougang Zhou, Huifeng Shao, Guojin Chen, Xiaowei Lin, Hongling Bie

**Affiliations:** 1 School of Mechanical Engineering, Hangzhou Dianzi University, Hangzhou 310018, China; 2 Ningbo Customs Technical Center, No. 8, Huikang Road, Yinzhou District, Ningbo 315800, China; 3 Mstar Technologies, Inc., Room 406, Building 19, Hangzhou Future Science and Technology City (Haichuang Park), No. 998, Wenyi West Road, Yuhang District, Hangzhou 311121, China; 4 Zhejiang Guangli Engineering Machinery Co., Ltd., No. 91, Guangda Street, Longquan, Lishui 323000, China; 5 Shanghai Institute of Electronic Information Technology, No. 30, Lane 24, Chezhan North Road, Hongkou District, Shanghai 200434, China

**Keywords:** platinum rhodium alloy, surface temperature distribution, semi-infinite lath structure, non-Fourier heat conduction equation, complex function method, conformal mapping method

## Abstract

In the fiberglass industry, Pt–Rh bushings made of platinum and rhodium have very good characteristics, such as high temperature resistance, corrosion resistance, oxidation resistance, and creep resistance. In this paper, a semi-infinite lath structure model is constructed, and the expression of the surface temperature distribution of a Pt–Rh alloy plate with a circular through hole is obtained based on the non-Fourier heat conduction equation, complex function method and conformal mapping method. At the same time, the influence of the position of the circular through hole in the Pt–Rh bushing and the parameters of the incident light source (Non-diffusion incident wave number and relative thermal diffusion length) on the surface temperature distribution of the Pt–Rh bushing is studied by using this formula. It is found that: 1. heat concentration and fracture are occur easily at the through hole; 2. when the through hole is in the asymmetric center, the greater the asymmetry, the smaller the maximum temperature amplitude; 3. when the buried depth of the through hole increases, the maximum temperature amplitude decreases; 4. when the incident wave number and the relative thermal diffusion length of the incident light source are larger, the maximum temperature amplitude is smaller. The numerical results are almost consistent with those of ANSYS thermal simulation. The expression of the surface temperature distribution of the semi-infinite lath structure proposed in this paper can effectively reduce the loss of precious metal materials and the time of thermal simulation in the experimental process, as well as provide important significance for structural design, quality inspection, process optimization, and service life improvement of Pt–Rh bushings.

## 1. Introduction

Rhodium is a very expensive precious metal. It is often called an industrial vitamin due to its high melting point, high strength, high temperature resistance, corrosion resistance, oxidation resistance, and catalysis [1]. Regarding the industrial application of rhodium, it is usually used as an additive element to improve the performance of other materials or metal materials in order to enhance their characteristics, and therefore, Rhodium plays an irreplaceable key role and is widely used in the fields of glass fiber and electronics.

Pt–Rh bushings made of platinum and rhodium are the most critical pieces of equipment in the glass fiber industry. During the operation of the Pt–Rh bushing, the electrodes at the two ends are connected to the circuit, the current through the bushing is as high as tens of kiloamperes, and the temperature inside the bushing is kept between 1100 °C and 1450 °C [2]. Although the Pt–Rh alloy has very high oxidation resistance and glass corrosion resistance, as well as good high-temperature mechanical properties, the Pt–Rh bushing is exposed to low stress, an oxidizing atmosphere, and the corrosive medium of high-temperature molten glass for a long time. In addition, the precious metal elements, such as Pt and Rh, in a bushing inevitably undergo oxidation and volatilization, which results in stress concentration and temperature field concentration near the round hole [3]. This leads to the reduction of the bearing capacity and service life of the Pt–Rh bushing. Therefore, it is of engineering significance to study the surface temperature distribution of the internal through holes of Pt–Rh bushings in a high temperature environment, which can provide a reference for the structural design, quality inspection, process optimization, and service life improvement of parts.

Fourier’s law is a basic law of heat transfer, expressed as:

(1) $$Q=-k\frac{dT}{dx}A$$

(2) $$q=\frac{Q}{A}$$

where, *Q* is the heat flux in W, *k* is the thermal conductivity in W/(m·K), *T* is the temperature in *K*, and x is the coordinate on the heat conducting surface in m, *A* is the cross-sectional area in m^2^, *q* is the heat flux density in W/m^2^.

According to Equation (1), the heat passing through a given cross-section in unit time is directly proportional to the temperature change rate and cross-section area in the direction perpendicular to the cross-section, while the direction of heat transfer is opposite to the direction of temperature increase [4].

In the classical Fourier heat conduction law, the default speed of heat conduction is infinite. That is, when the temperature changes at a certain point inside an object, it can instantly cause the temperature changes at other positions. However, in fact, the propagation speed of heat conduction in an object is limited, and therefore, the traditional Fourier heat conduction law is only applicable to the steady heat conduction problem with long heat diffusion time, and the conventional unsteady heat conduction problem with short heat diffusion time [5].

With the rapid development of science and technology, heat conduction environments are becoming more and more extreme, such as ultra-high temperature and ultra-low temperature conditions, femtosecond time and nanoscale space scale, pulse laser heating and other high-frequency and high-heat conditions. However, the traditional Fourier heat conduction law does not consider the time factor, which is referred to as the non-Fourier effect [6].

In order to overcome the limitations of the Fourier law, many studies tackled the non-Fourier heat conduction law. For instance, Cattaneo [7] and Vernotte [8] treated heat conduction as a process, and thought that the thermal diffusion in the medium took time. Thus, they introduced the concept of thermal relaxation time, that is, the time when the temperature field in the material reaches a stable state again through thermal propagation when the temperature changes. The modified differential equation of heat conduction is expressed as:

(3) $$k_{d}\nabla^{2}T=\frac{\partial T}{\partial t}+\tau_{q}\frac{\partial^{2}T}{\partial t^{2}}$$

Compared with the traditional Fourier heat conduction model, the non-Fourier heat conduction model proposed by Cattaneo and Vernotte changes from a parabolic differential equation to a hyperbolic differential equation in time and space. Therefore, the non-Fourier heat conduction model in Equation (2) is also referred to as a hyperbolic heat wave model or single-phase delay model (CV model). This model can describe the heat wave behavior in extreme heat conduction, and its mathematical form is concise; not much more complicated than the classical Fourier model. Therefore, the CV model has been widely studied, and it is a non-Fourier heat conduction model with great influence.

Tzou [9] considered that there was a time delay between heat flow propagation and temperature gradient formation from a macroscopic point of view. He developed heat conduction models of single-phase delay and two-phase delay. Zhao Weitao et al. [10] used the Fourier series expansion method and superposition principle to obtain the analytical expression of temperature field under non-Fourier heat conduction, when the heat flux on the front surface of a flat plate randomly changes. Sarkar and HajiSheikh [11] studied the hyperbolic heat conduction of finite plates made of dielectric materials by Laplace transform. Tang and Araki [12] solved the problem of non-Fourier heat conduction of finite medium under periodic surface heating. Li Shirong et al. [13] studied the thermal response of thin plate under periodic heat flow based on single-phase hyperbolic non-Fourier heat conduction equation.

Li Jine [14] studied the non-Fourier fracture performance of solid plate under thermal shock, and analyzed the impacts of the relaxation time, crack length, and material thickness on the thermal stress intensity factor. By comparing the thermal stress intensity factors under non-Fourier thermal shock with those under classical thermal shock, they deduced that the smaller the thickness of the solid plate, the more obvious the non-Fourier effect. Lu and Fleck [15] studied the one-dimensional thermal shock fracture of thin plate structures. Noda and Jin [16,17,18,19] studied the fracture of functionally graded strip plates and functionally graded semi-infinite plates under static thermal load and impact thermal load, respectively. Guo Songlin [20] used the two-phase delayed non-Fourier heat conduction model, and applied the integral transformation method and singular integral equation to solve the thermal shock fracture problem of plate structures in extreme environments such as ultra-high temperature, ultra-low temperature, and micro-scale.

Zhang Shiyuan et al. [21] used the temperature field obtained by the non-Fourier heat conduction model as the thermal load. They analyzed the driving force of the unilateral crack of the thermal coating using the finite element method. G.V. Kolosov and N.I. Muskhelishvili [22] solved the stress concentration problem of arbitrarily shaped holes using complex function and conformal mapping method. Nied and Erdogan [23] studied the one-dimensional thermal shock of a hollow cylinder with a circumferential crack. Fu et al. [24] studied the non-Fourier thermoelastic behavior of a hollow cylinder with an embedded circumferential crack. Chen and Hu [25,26,27] studied the temperature response of a thermoelastic plate with an internal crack parallel to the boundary under the temperature impact load. They obtained the temperature around the crack of the substrate bonded with the coating using the hyperbolic heat conduction model.

Previous studies on heat conduction in plate-like structures have been very mature. In addition, the thermal shock fracture of plate-like structures has been further studied, which provides a basis for the follow-up studies on the influence of through holes on heat conduction in plate-like structures. However, at present, most of the existing studies are based on cracks (that is, the influence of line defects on the internal heat conduction of plate-like structures), and the position and direction of these cracks are mostly at the edge or parallel to the boundary, which presents certain limitations. Moreover, cracks are a kind of surface defect that appears in the process of machining. Therefore, it is difficult to reasonably and effectively optimize the appearance and size of the cracks. Some researchers have also studied the response of arbitrarily shaped holes in infinite plate structures to temperature distribution under temperature shock load. However, the heat exchange phenomenon at the boundary is neglected in the infinite plate structure, which is not closely related to the actual generation and application.

A Pt–Rh bushing is a kind of precious metal material structure with a circular through hole that is applied in a high temperature environment. In this paper, a semi-infinite lath structure model is built for a Pt–Rh bushing. Based on the non-Fourier heat conduction equation, complex function method and conformal mapping method, the surface temperature distribution expression of a Pt–Rh bushing with a circular through hole is obtained. By controlling the position of the circular through hole and the frequency and amplitude of the laser pulse, the surface temperature distribution of the round through hole in the Pt–Rh bushing at different positions, and when the incident light source adopts different non-diffusion incident wave numbers and relative thermal diffusion lengths, is obtained. The results are almost consistent with the thermal simulation results of ANSYS. According to the surface temperature distribution expression of semi-infinite lath structure proposed in this paper, the optimal range of experimental parameters can be determined efficiently before the experiment and simulation, which can effectively reduce the loss of precious metal materials and the time of thermal simulation in the experimental process. This has important reference significance for the structural design, quality inspection, process optimization and service life improvement of Pt–Rh bushings, and also provides a certain reference value for other related research of metal materials with perforated plate structures.

## 2. Materials and Methods

**Figure 1:**
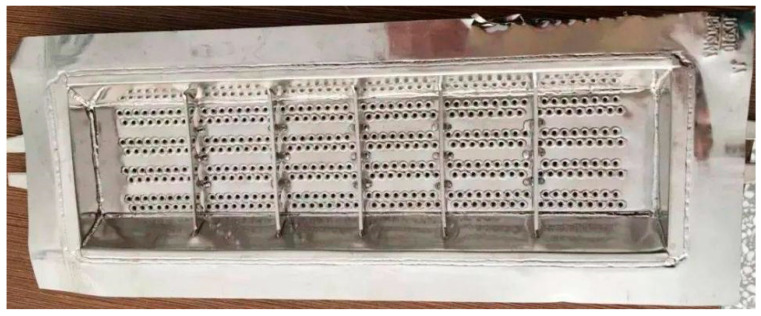
Pt–Rh bushing.

There are many circular through holes evenly distributed on the Pt–Rh bushing, as shown in Figure 1.

According to the research of Pt–Rh alloy by Wuxi Indium Metal Products Co., Ltd. (Wuxi, China), the related parameters of Pt–Rh alloy with a rhodium content of 10% (Table 1) can be obtained [28].

**Figure 2:**
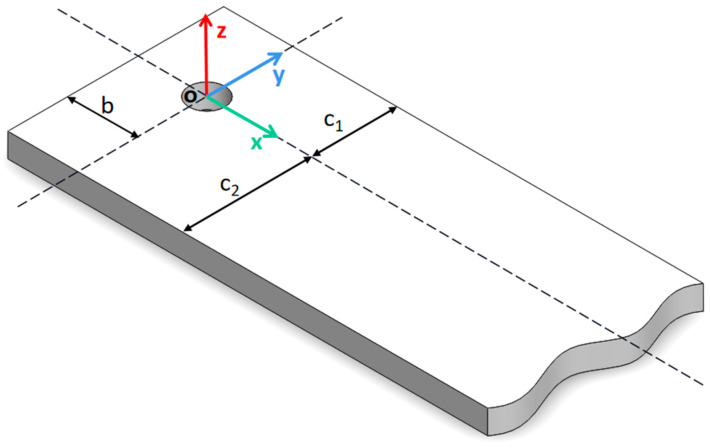
Schematic diagram of the three-dimensional object structure.

In order to further study the influence of the position of the circular through hole in the Pt–Rh bushing and the wave number of the incident light source on the surface temperature distribution, the Pt–Rh bushing structure is considered equivalent to the semi-infinite slab structure shown in Figure 2 (that is, the boundary conditions on the right are not discussed), the circular through hole is considered equivalent to the adiabatic cylinder with radius *a* buried in the semi-infinite slab structure, the distance from the left surface is taken as *b*, the distance from the upper surface is taken as *c*_1_, and the distance from the lower surface is taken as *c*_2_.

The laser pulse beam with modulation frequency *f* is irradiated on the outer surface of the Pt–Rh bushing from the left side, and a thermal wave is formed inside it. Assuming that the distribution of temperature field in the *Z*-axis direction is uniform, this research will be transformed into the study of the two-dimensional temperature field distribution in the coordinate system (*x*, *y*) on the semi-infinite slab structure of Pt–Rh alloy.

## 3. The Wave Equation of Heat Conduction and Its General Solution

The temperature control equation in solid medium without internal heat source (Equation (4)) can be obtained according to the non-Fourier heat conduction equation [29,30,31]:

(4) $$\nabla^{2}T=\frac{1}{D}\frac{\partial T}{\partial t}+\frac{1}{c^{2}}\frac{\partial^{2}T}{\partial t^{2}}$$

(5) $$\nabla^{2}=\frac{\partial^{2}}{\partial x^{2}}+\frac{\partial^{2}}{\partial y^{2}}$$

(6) $$D=\frac{\lambda }{\rho c_{p}}$$

(7) $$c=\sqrt{D/\tau }$$

where, $\nabla^{2}$ is the Laplace operator, *D* is the thermal diffusivity, *c* is the thermal wave propagation speed, λ is the thermal conductivity of the material, *c_p_* is the specific heat capacity of the material at constant pressure, *ρ* is the density of material, *τ* is the thermal relaxation time, and *T* is the temperature in solid medium.

Regarding the wave problem, it can be analyzed and solved by the time domain solution and frequency domain solution, and the above two solutions can be converted by Fourier transform. The nondestructive testing of the through holes in the material can be performed by applying periodic thermal stimulation to the material so that the position, orientation, and size of the through holes in the material can be obtained.

According to the Fourier decomposition theorem, the periodic heat conduction process can be considered as the superposition of several simple harmonic vibrations. Therefore, the periodic unsteady heat conduction can be studied according to the wave theory.

According to the periodic unsteady heat conduction solution of Equation (4), the temperature field can be expressed as:

(8) T=Tm+Reϑexp−iωt

where, the temperature amplitude ϑ should satisfy the Helmholtz equation in the following form [32]:

(9) $$\nabla^{2}\vartheta +\kappa^{2}\vartheta =0$$

(10) $$\vartheta =T_{max}-T_{m}$$

(11) $$\nabla^{2}=\frac{\partial^{2}}{\partial x^{2}}+\frac{\partial^{2}}{\partial y^{2}}$$

(12) $$\kappa =\sqrt{\left(\frac{\omega^{2}}{c^{2}}+i\frac{\omega }{D}\right)}=\alpha +i\beta$$

where, Re is the real part, *T_m_* is the ambient average temperature, ϑ is the excess temperature amplitude, $\nabla^{2}$ is the Laplace operator, *ω* is the circular frequency, κ is the complex variable wave number, i is the imaginary unit, *α* is the wave number of thermal wave propagation, and β is the thermal wave absorption coefficient.

After normalizing α and β, Equations (13) and (14) can be obtained:

(13) $$\alpha =\sqrt{\frac{1}{2}\left[\sqrt{\frac{\omega^{4}}{c^{4}}+\frac{\omega^{2}}{D^{2}}}+\frac{\omega^{2}}{c^{2}}\right]}=\sqrt{\sqrt{\frac{1}{4}k^{2}+\frac{1}{\mu^{4}}}+\frac{1}{2}k^{2}}\;\;\;\;\;\mathrm{and}\;\alpha >0$$

(14) $$\beta =\sqrt{\frac{1}{2}\left[\sqrt{\frac{\omega^{4}}{c^{4}}+\frac{\omega^{2}}{D^{2}}}-\frac{\omega^{2}}{c^{2}}\right]}=\sqrt{\sqrt{\frac{1}{4}k^{2}+\frac{1}{\mu^{4}}}-\frac{1}{2}k^{2}}\;\;\;\;\;\mathrm{and}\;\beta >0$$

where, *µ* is the thermal diffusion length, *k* = *ω*/*c* is the number of thermal waves without diffusion effect, *ω* is the circular frequency, and *c* is the propagation speed of thermal waves.

When the heat wave propagation velocity *c* approaches infinity, the non-Fourier heat conduction degenerates into the classical Fourier heat diffusion propagation. At this time, both α and β approach 1/μ. In addition, κ approaches $\left(1+i\right)/\mu$. It can be seen that at this time, there is a waveform shown in Equation (15) in the solid medium, which represents the propagating wave with attenuation of vibration amplitude in space [32].

(15) $$\vartheta e^{-i\omega t}=Ae^{-\beta x}e^{i\left(\alpha x-\omega t\right)}$$

The complex variable shown in Equation (16) can be introduced using the method of complex variable function:

(16) $$z=x+iy\;,\bar{z}=x-iy$$

Equation (17) can then be obtained:

(17) $$x=\left(z+\bar{z}\right)/2\;\;,y=\left(z-\bar{z}\right)/2i$$

Equation (9) can be reduced to the following form:

(18) $$\frac{\partial^{2}\vartheta }{\partial z\partial \bar{z}}+{\left(\frac{\kappa }{2}\right)}^{2}\vartheta =0$$

The general solution of the thermal wave scattering field of a single circular through hole in a semi-infinite solid medium, determined by Equation (9), is given by [32]:

(19) $$\vartheta =\overset{\infty }{\underset{n=-\infty }{\sum }}A_{n}H_{n}^{\left(1\right)}\left(\kappa r\right)e^{-in\theta }=\overset{\infty }{\underset{n=-\infty }{\sum }}A_{n}H_{n}^{\left(1\right)}\left(\kappa \left|z\right|\right){\left\{\frac{z}{\left|z\right|}\right\}}^{n}$$

where, $A_{n}$ is the scattering wave mode coefficient determined by the boundary conditions of the through hole and $H_{n}^{\left(1\right)}\left(\cdot \right)$ is the Hankel function of the first kind with complex variables.

## 4. General Solution of Heat Wave Scattering by Subsurface through Holes in Lath Structure

The conformal mapping method can be used to solve the plane wave scattering problem of a through hole with an arbitrary shape in a Pt–Rh bushing. The mapping function of the circular via boundary on the *z* plane to the unit circle boundary on the *y* plane can be expressed as [28]:

(20) $$z=\Omega \left(\zeta \right)$$

At this time, there is $z=re^{i\varphi }$ on the z plane and polar coordinates are used on the mapping plane ζ. Therefore, the coordinates of any point can be expressed as $\zeta =\rho e^{i\theta }$ on the ζ plane. Thus, the general solution of the thermal wave scattering field of a single circular through hole in the Z plane can be expressed as [32]:

(21) $$\vartheta =\overset{\infty }{\underset{n=-\infty }{\sum }}A_{n}H_{n}^{\left(1\right)}\left(\kappa \left|\Omega \left(\zeta \right)\right|\right){\left\{\frac{\Omega \left(\zeta \right)}{\left|\Omega \left(\zeta \right)\right|}\right\}}^{n}$$

For a circular through hole with diameter *A*, the guaranteed angle mapping function is given by:

(22) $$z=\Omega \left(\zeta \right)=a\left(\zeta +\frac{\varepsilon }{\zeta }\right)$$

## 5. Excitation of Incident Wave and Total Wave Field

In this paper, it is assumed that an ultrashort laser pulse source heats the semi-infinite slab structure of Pt–Rh bushing from the left side, and the temperature wave propagates along the positive X direction. Based on the constructive interference theory of wave field, the expression of temperature distribution in the semi-infinite strip structure of Pt–Rh bushing can be set as follows [32]:

(23) $$\vartheta =f\left(y\right)\exp\left[i\left(px-\omega t\right)\right]$$

The following formula can be obtained by combining vertical (23) with Equation (8):

(24) $$f\left(y\right)=A\cos\left(qy\right)+B\sin\left(qy\right)$$

(25) $$p^{2}=\kappa^{2}-q^{2}$$

where, *p* is the longitudinal wave number of temperature fluctuation, and *q* is the transverse wave number of temperature fluctuation.

The temperature boundary conditions of the upper and lower surfaces are given by:

(26) $$f\left(c_{1}\right)\exp\left(ipx\right)=0$$

(27) $$f\left(-c_{2}\right)\exp\left(ipx\right)=0$$

The transverse wave number of temperature fluctuation can then be obtained:

(28) $$q=\frac{n\pi }{c_{1}+c_{2}}\;\;\;\;\;\;\;\;\;\;\;\;\;\;\;\left(n=0,1,2,\cdots \infty \right)$$

By combining Vertical (28) and Equation (18), the expression of temperature distribution in solid medium can be obtained:

(29) $$\vartheta =B\frac{\sin\left[q\left(c_{2}+y\right)\right]}{\cos\left(qc_{2}\right)}\;\exp\left[i\left(px-\omega t\right)\right]$$

In this paper, it is assumed that a periodic steady-state heat wave is incident in the positive direction of the *X*-axis at the semi-infinite slab structure of the Pt–Rh bushing, and the temperature wave propagates in the positive direction of *x*. The mirror method can then be used to consider the reflected wave at the boundary of a semi-infinite strip, and thus, study the incident wave.

When the upper and lower surfaces are consistent with the ambient temperature, the temperature wave can be expressed as [32]:

(30) $$\vartheta =\vartheta_{0}sin\left[q\left(c_{2}+y\right)\right]=\vartheta_{0}sin\left[q\left(c_{2}+y\right)\right]e^{ipb}\overset{\infty }{\underset{n=-\infty }{\sum }}i^{n}J_{n}\left(pr\right)e^{in\theta }e^{-i\omega t}$$

(31) $$\vartheta_{0}=T_{0}-T_{m}$$

(32) $$q=\frac{\pi }{c_{1}+c_{2}}$$

where, $\vartheta_{0}$ is the temperature amplitude of the incident heat wave (i.e., the excess temperature), *p* is the wave number of the propagating wave along the *X*-axis, and $J_{n}\left(\cdot \right)$ is the Bessel function.

In the case where the upper and lower surfaces have no heat dissipation from the environment, the temperature wave is expressed as:

(33) $$\vartheta^{\left(i\right)}=\vartheta_{0}e^{ip\left(x+b\right)}e^{-i\omega t}$$

Considering the multiple scattering effects of $y=c_{1}$, $y=c_{1}$, and x=−b at the boundary of a semi-infinite slab, the thermal wave scattering field generated by a single circular through hole in polar coordinate system can be expressed as:

(34) $$\begin{array}{l} \vartheta^{\left(s\right)}=\overset{\infty }{\underset{n=-\infty }{\sum }}A_{n}H_{n}^{\left(1\right)}\left(\kappa r\right)e^{in\varphi }+\overset{\infty }{\underset{n=-\infty }{\sum }}A_{n}{\left(-1\right)}^{n}H_{n}^{\left(1\right)}\left(\kappa r^{\prime }\right)e^{in\varphi^{\prime }}\\ +\overset{\infty }{\underset{m=1}{\sum }}\left\{\overset{\infty }{\underset{n=-\infty }{\sum }}A_{n}H_{n}^{\left(1\right)}\left(\kappa r_{m}\right)e^{-in\varphi_{m}}+\overset{\infty }{\underset{n=-\infty }{\sum }}A_{n}{\left(-1\right)}^{n}H_{n}^{\left(1\right)}\left(\kappa {r^{\prime }}_{m}\right)e^{in{\varphi^{\prime }}_{m}}\right\} \end{array}$$

The complex variable form solution of the thermal wave scattering field of an arbitrary through hole on the $\zeta =\rho e^{i\theta }$ plane is given by:

(35) $$\begin{array}{ll} \vartheta^{\left(s\right)}= & \overset{\infty }{\underset{n=-\infty }{\sum }}A_{n}H_{n}^{\left(1\right)}\left(\kappa \left|z\right|\right){\left\{\frac{z}{\left|z\right|}\right\}}^{n}+\overset{\infty }{\underset{n=-\infty }{\sum }}A_{n}{\left(-1\right)}^{n}H_{n}^{\left(1\right)}\left(\kappa \left|z-z_{0}\right|\right){\left\{\frac{z-z_{0}}{\left|z-z_{0}\right|}\right\}}^{-n}\\ & +\overset{\infty }{\underset{n=-\infty }{\sum }}A_{n}\overset{4}{\underset{l=1}{\sum }}\overset{\infty }{\underset{m=1}{\sum }}H_{n}^{\left(1\right)}\left(\kappa \left|z-z_{lm}\right|\right){\left\{\frac{z-z_{lm}}{\left|z-z_{lm}\right|}\right\}}^{{\left(-1\right)}^{l}n}\;\;\;\;\;\;\;\;\;\;\;\;\;\;\;\;\;\;\;\;\;\\ & +\overset{\infty }{\underset{n=-\infty }{\sum }}A_{n}{\left(-1\right)}^{n}\overset{4}{\underset{l=1}{\sum }}\overset{\infty }{\underset{m=1}{\sum }}H_{n}^{\left(1\right)}\left(\kappa \left|z-z_{0}-z_{lm}\right|\right){\left\{\frac{z-z_{0}-z_{lm}}{\left|z-z_{0}-z_{lm}\right|}\right\}}^{{\left(-1\right)}^{l+1}n} \end{array}$$

where, $z_{0}=-2b,\;z_{1m}=2i\left(mL-c_{2}\right),\;z_{2m}=2imL,\;z_{3m}=-i2\left[\left(m-1\right)L+c_{2}\right],\;z_{4m}=-2imL,\;L=c_{1}+c_{2},m=1,2,3\ldots \infty ,\;r=\left|\Omega \left(\zeta \right)\right|,\;\mathrm{and}\;z=\Omega \left(\zeta \right),\;z^{\prime }=\Omega^{\prime }\left(\zeta \right).$

The total field of thermal wave in solid medium should be composed of incident wave and scattered wave, and it is expressed as:

(36) $$\vartheta^{\left(t\right)}=\vartheta^{\left(i\right)}+\vartheta^{\left(s\right)}$$

## 6. Determination of Thermal Wave Mode Coefficient

The infinite algebraic equations for determining *A_n_* shown in Equation (37), can be obtained by substituting Equation (36) into the temperature expression (Equation (22)) on the via boundary condition (*ρ* = 1):

(37) $$\overset{\infty }{\underset{n=-\infty }{\sum }}\in_{n}X_{n}=\in$$

(38)     ∈n=12κReζz′ z¯ζz[Hn−11κz−Hn+11κz]zzn+niImζz′ζzHn1κzzzn            +12κ−1nReζz′z¯−z0¯ζz−z0[Hn−11κz−z0−Hn+11κz−z0]z−z0z−z0−n  +ni−1n+1Imζz′ζz−z0Hn1κz−z0z−z0z−z0−n                                         +12κ∑m=1∞∑l=14Reζz′z¯−zlm¯ζz−zlm[Hn−11κz−zlm−Hn+11κz−zlm]z−zlmz−zlm−1ln+ni∑m=1∞∑l=14−1lImζz′ζz−zlmHn1κz−zlmz−zlmz−zlm−1ln+12κ∑m=1∞∑l=14−1nReζz′z¯−z0¯−zlm¯ζz−z0−zlm[Hn−11κz−z0−zlm     −Hn+11κz−z0−zlm]z−z0−zlmz−z0−zlm−1l+1n      +ni∑m=1∞∑l=14−1n+l+1Imζz′ζz−z0−zlmHn1κz−z0−zlmz−z0−zlmz−z0−zlm−1l+1n

(39) $$\in =-\vartheta_{0}\left\{q\sin\varphi \cos\left[q\left(c_{2}+y\right)\right]+ip\cos\varphi \sin\left[q\left(c_{2}+y\right)\right]\right\}\exp\left[ip\left(x+b\right)\right]$$

(40) z=reiφ, z=Ωζ, ζ=ρeiθ, cosφ=Rezz, sinφ=Imzz, Xn=An

where, *Re* represents the real part and Im represents the imaginary part.

$e^{-is\theta }$ is then multiplied by the two ends of Equation (37) to obtain the infinite algebraic equations:

(41) $$\overset{\infty }{\underset{n=-\infty }{\sum }}\in_{ns}X_{n}=\in_{s}\;\;\;\;\;\;\;\left(n=s=0,\pm 1,\pm 2\cdots \right)$$

By integrating over the interval (−π,+π), the following formula can be obtained:

(42) $$\in_{ns}=\frac{1}{2\pi }\int_{-\pi }^{\pi }\in_{n}e^{-is\theta }d\theta$$

(43) $$\in_{s}=\frac{1}{2\pi }\int_{-\pi }^{\pi }\in e^{-is\theta }d\theta$$

Equation (41) represents the infinite algebraic equations for determining the thermal wave scattering mode coefficient *A_n_*.

When an object contains a through hole, multiple scattering of heat waves will occur between the through hole and the surface, which will affect the temperature of the object surface. Periodic heating will produce temperature fluctuation in the object, and thermal wave imaging can be performed using the temperature amplitude change and phase difference caused by through holes. By measuring the change of the surface temperature, the through-holes on the surface of the object can be perceived.

The radius of the circular through hole on the Pt–Rh bushing is *a*, and the incident amplitude of the excess temperature is $\left|\vartheta_{0}\right|$. The following dimensionless quantities are used in the calculation: the non-diffusion incident wave number *ka*, the relative thermal diffusion length μ/a, the buried depth ratio b/a (when b/a is less than 1, the circular through hole interferes with the left edge of the Pt–Rh bushing, and when the buried depth ratio b/a is too large, it is equivalent to an infinite slab structure, and thus, b/a=1.1~2.0 is defined in this paper), the distance ratio $c_{1}/a$ and $c_{2}/a$ between the circular through hole and the upper and lower surfaces, and the excess temperature ratio $\vartheta /\vartheta_{0}$, so that the dimensionless complex number can be obtained:

(44) κa=αa+iβa

(45) $$\alpha a=\sqrt{\sqrt{\frac{1}{4}{\left(ka\right)}^{4}+{\left(\frac{a}{\mu }\right)}^{4}}+\frac{1}{2{\left(ka\right)}^{2}}}$$

(46) $$\beta a=\alpha a=\sqrt{\sqrt{\frac{1}{4}{\left(ka\right)}^{4}+{\left(\frac{a}{\mu }\right)}^{4}}-\frac{1}{2{\left(ka\right)}^{2}}}$$

The mathematical expression of the surface temperature distribution of the measured object can then be obtained:

(47) $$\begin{array}{l} \;\vartheta =\vartheta_{0}\sin\left[q\left(c_{2}+y\right)\right]\exp\left[ip\left(x+b\right)\right]+\overset{\infty }{\underset{n=-\infty }{\sum }}A_{n}H_{n}^{\left(1\right)}\left(\kappa \left|z\right|\right){\left\{\frac{z}{\left|z\right|}\right\}}^{n}\\ \;+\overset{\infty }{\underset{n=-\infty }{\sum }}A_{n}{\left(-1\right)}^{n}H_{n}^{\left(1\right)}\left(\kappa \left|z-z_{0}\right|\right){\left\{\frac{z-z_{0}}{\left|z-z_{0}\right|}\right\}}^{-n}\\ \;+\overset{\infty }{\underset{n=-\infty }{\sum }}A_{n}\overset{4}{\underset{l=1}{\sum }}\overset{\infty }{\underset{m=1}{\sum }}\left[H_{n}^{\left(1\right)}\left(\kappa \left|z-z_{lm}\right|\right){\left\{\frac{z-z_{lm}}{\left|z-z_{lm}\right|}\right\}}^{{\left(-1\right)}^{l}n}\right]\\ +\overset{\infty }{\underset{n=-\infty }{\sum }}A_{n}\overset{4}{\underset{l=1}{\sum }}\overset{\infty }{\underset{m=1}{\sum }}\left[{\left(-1\right)}^{n}H_{n}^{\left(1\right)}\left(\kappa \left|z-z_{0}-z_{lm}\right|\right){\left\{\frac{z-z_{0}-z_{lm}}{\left|z-z_{0}-z_{lm}\right|}\right\}}^{{\left(-1\right)}^{l+1}n}\right] \end{array}$$

where, x=−b, y=0~4a, and z−b+iy

## 7. Numerical Examples

**Figure 3:**
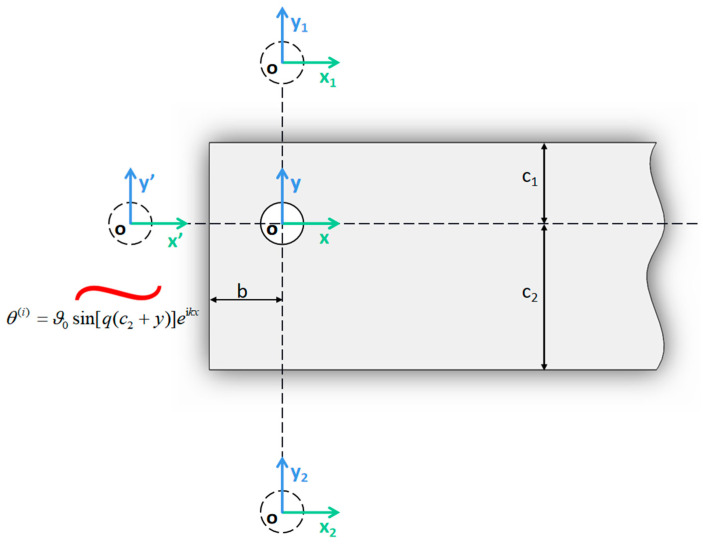
Schematic diagram of two-dimensional pulse heating.

According to the schematic diagram of two-dimensional pulse heating shown in Figure 3, a plane coordinate system is established with the center of a circular through hole as the origin, the horizontal right direction as the positive direction of the *X*-axis, and the vertical upward direction as the positive direction of the *Y*-axis.

**Figure 4:**
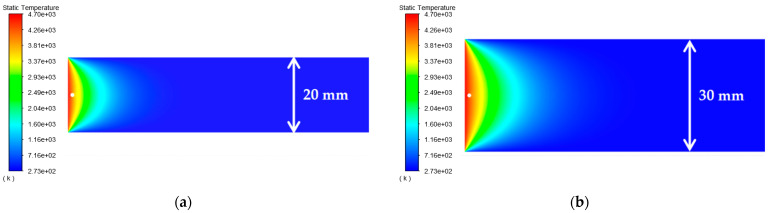

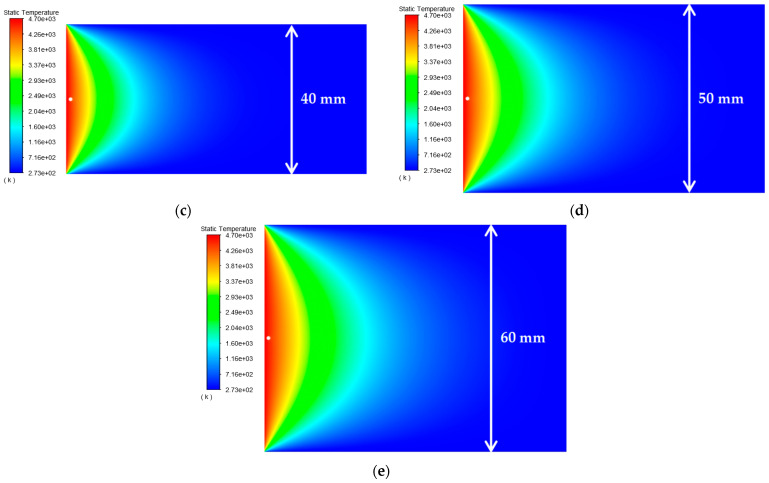
Surface temperature distribution diagram of platinum rhodium bushing: (**a**) *c*_1_ = *c*_2_ = 10 mm; (**b**) *c*_1_ = *c*_2_ = 15 mm; (**c**) *c*_1_ = *c*_2_ = 20 mm; (**d**) *c*_1_ = *c*_2_ = 25 mm; (**e**) *c*_1_ = *c*_2_ = 30 mm.

**Figure 5:**
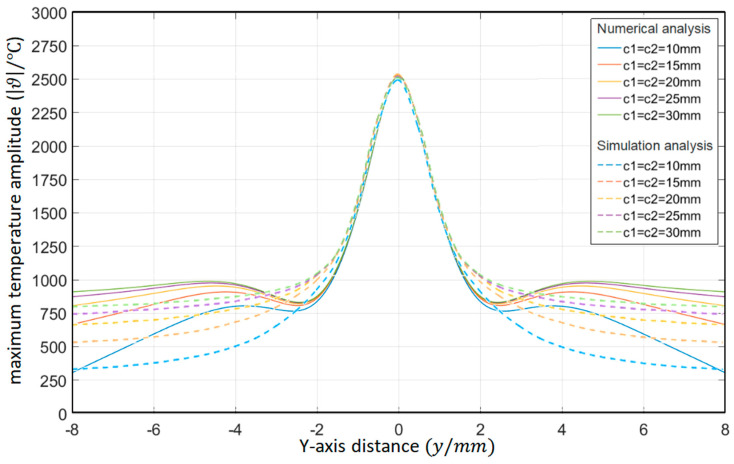
Influence of the distance from the circular through hole to the upper and lower surfaces of the Pt–Rh bushing on the temperature distribution in the *Y*-axis direction.

The temperature amplitude of the incident heat wave is then given as 1300 °C, then the position of the circular through hole and the parameters of the incident light source are changed, and the temperature distribution results are obtained by the above-mentioned temperature distribution expression. At the same time, the corresponding thermal simulation analysis is carried out by ANSYS, and the two analysis results are compared to verify the rationality of the semi-infinite slab structure model constructed in this paper and the accuracy of the temperature distribution expression. The simulation contents and results are as follows:

As shown in Table 2, the distance between the circular through hole and the upper and lower surfaces of the platinum-rhodium bushing is *c*_1_ = *c*_2_ = 10 mm, 15 mm, 20 mm, 25 mm, and 30 mm, respectively. The surface temperature distribution diagram shown in Figure 4 and the “*y*-$\left|\vartheta \right|$” diagram shown in Figure 5 can be obtained by ANSYS 2020 R2 and MATLAB R2020b.

According to the results in Figure 5, their temperature change trends are almost the same. When the distances from the circular through hole to the upper and lower surfaces of the Pt–Rh bushing are equal, the peak temperature of the object surface appears in front of the circular through hole, and the temperature change curve is symmetrical about y = 0. When the distances *c_1_* and *c_2_* between the circular through hole and the upper and lower surfaces increase synchronously, the peak temperature hardly changes. In *y*∈(−8, −2) and *y*∈(2, 8), with the gradual increase of *c*_1_ and *c*_2_, the temperature change rate clearly slows down. This is because the distance between the circular through hole and the upper and lower surfaces of the Pt–Rh bushing decreases the heat exchange with the external environment, and therefore, the temperature changes relatively slowly.

**Figure 6:**
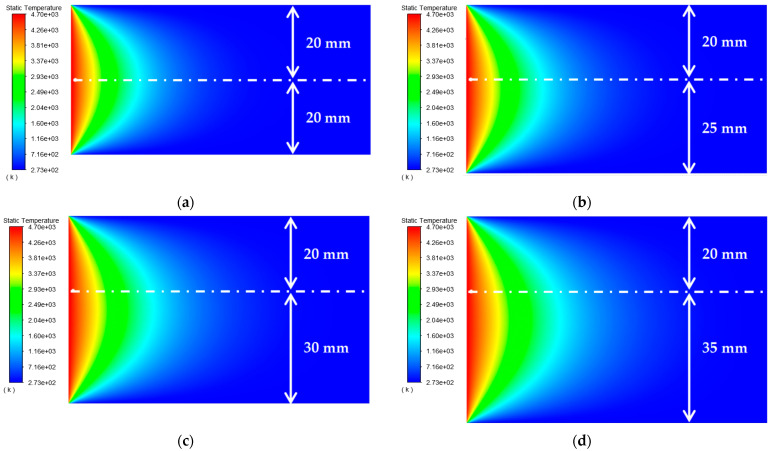

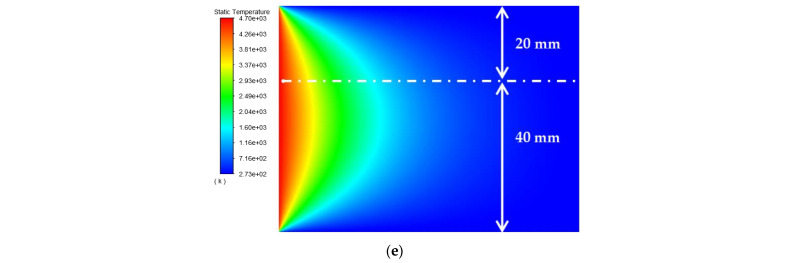
Surface temperature distribution diagram of platinum rhodium bushing: (**a**) *c*_2_ = 20 mm; (**b**) *c*_2_ = 25 mm; (**c**) *c*_2_ = 30 mm; (**d**) *c*_2_ = 35 mm; (**e**) *c*_2_ = 30 mm.

**Figure 7:**
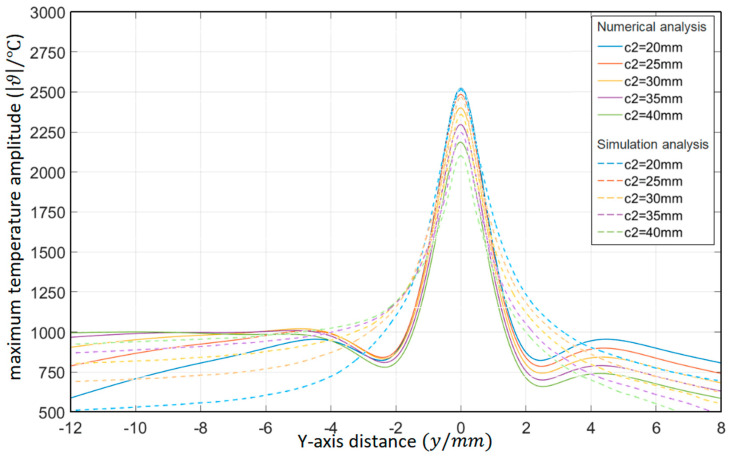
Influence of the distance from the circular through hole to the single surface of the Pt–Rh bushing on the temperature distribution in the *Y*-axis direction.

As shown in Table 3, the distance between the circular through hole and the lower surfaces of the platinum-rhodium bushing is *c*_2_ = 20 mm, 25 mm, 30 mm, 35 mm, and 40 mm, respectively. The surface temperature distribution diagram shown in Figure 6 and the “*y*-$\left|\vartheta \right|$” diagram shown in Figure 7 can be obtained by ANSYS and MATLAB.

According to the results in Figure 7, their temperature change trends are almost the same. When the distance between the circular through hole and the upper and lower surfaces of the Pt–Rh bushing is not equal, the peak temperature of the object surface still appears in front of the circular through hole, but the temperature change curve is no longer symmetrical; about *y* = 0. When the distance ratio *c*_1_/*a* between the circular through hole and the upper surface of the Pt–Rh bushing remains constant and the distance *c_2_* between the circular through hole and the lower surface gradually increases, the peak temperature will decrease with the increase of *c_2_*. In *y*∈(−2,8), when the distance *c_2_* between the circular through hole and the lower surface is larger, the temperature is lower. In *y*∈(−12, −6), the larger the distance *c_2_* between the circular through hole and the lower surface, the higher the temperature and the slower the temperature change. When opening a hole on the Pt–Rh bushing, the circular through hole can be set at the center line deviated from the *Y*-axis direction so as to avoid the excessive superimposed temperature at the circular through hole.

**Figure 8:**
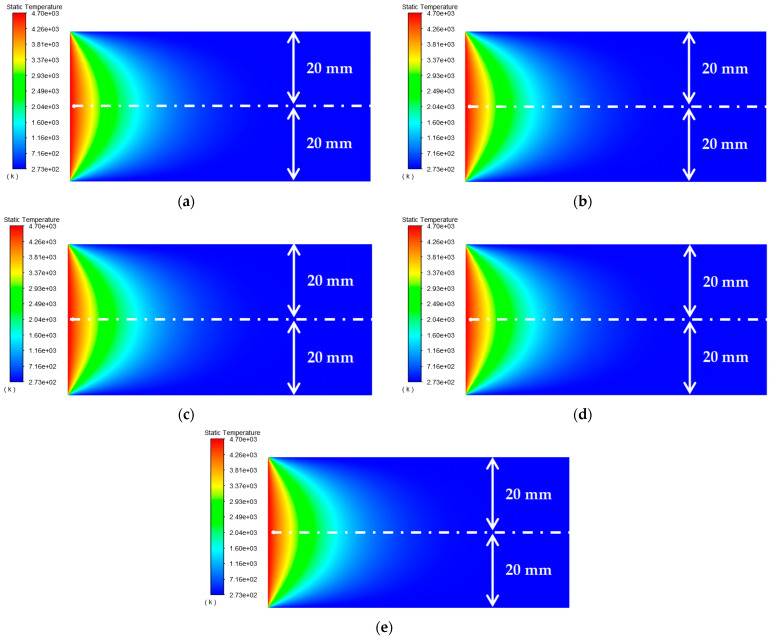
Surface temperature distribution diagram of platinum rhodium bushing: (**a**) *b* = 1.1 mm; (**b**) *b* = 1.2 mm; (**c**) *b* = 1.3 mm; (**d**) *b* = 1.4 mm; (**e**) *b* = 1.5 mm.

**Figure 9:**
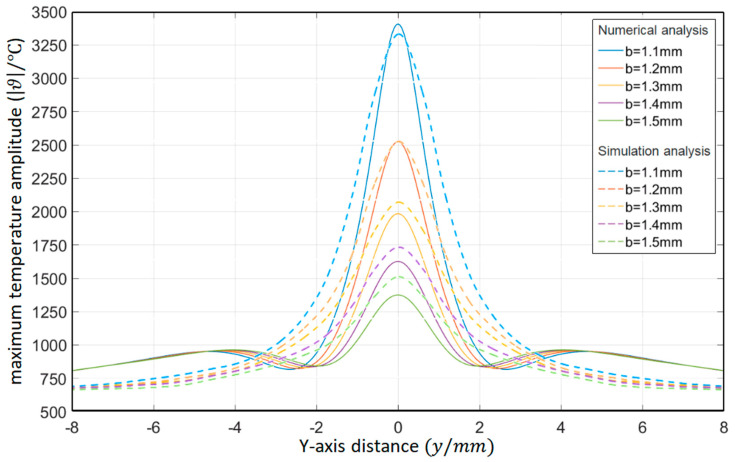
Influence of the buried depth of circular through hole on the temperature distribution in the *Y*-axis direction.

As shown in Table 4, the buried depth of the circular through hole is *b* = 1.1 mm, 1.2 mm, 1.3 mm, 1.4 mm, and 1.5 mm, respectively. The surface temperature distribution diagram shown in Figure 8 and the “y-$\left|\vartheta \right|$” diagram shown in Figure 9 can be obtained by ANSYS and MATLAB.

According to the results in Figure 9, their temperature change trends are almost the same. When *b* gradually increases, the peak temperature gradually decreases, and when the buried depth is large enough, the temperature concentration tends to disappear. When opening a hole in the Pt–Rh bushing, the buried depth of the circular through hole should not be too shallow.

**Figure 10:**
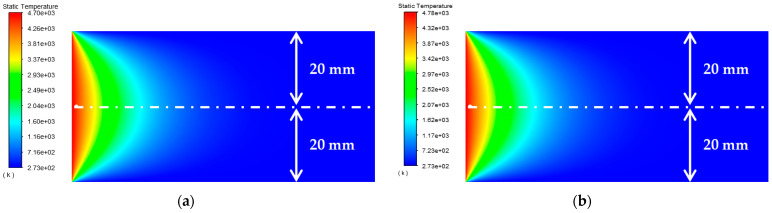

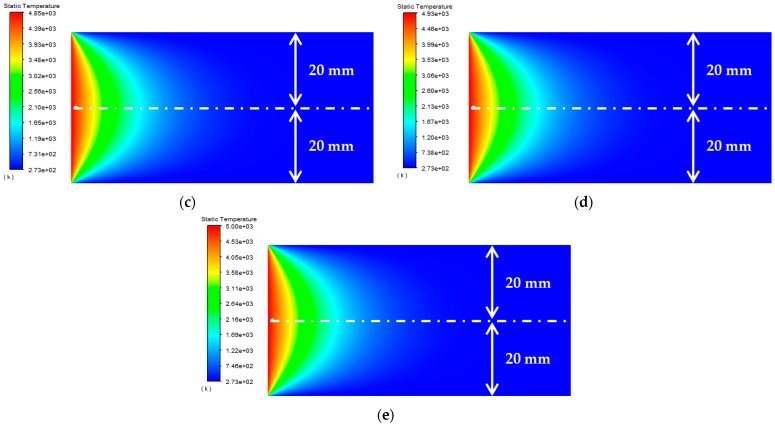
Surface temperature distribution diagram of platinum rhodium bushing: (**a**) *ka* = 1.1; (**b**) *ka* = 1.1; (**c**) *ka* = 1.1; (**d**) *ka* = 1.1; (**e**) *ka* = 1.1.

**Figure 11:**
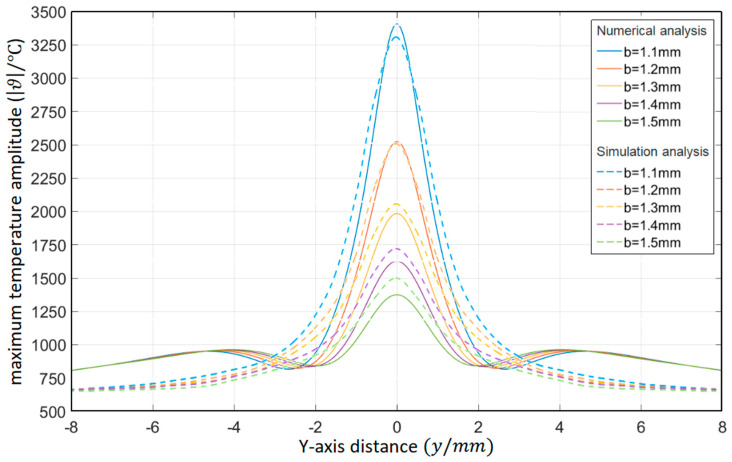
Influence of the non-diffusion incident wave number on the temperature distribution in the *Y*-axis direction).

As shown in Table 5, the non-diffusion incident wave number is *ka* = 1.1, 1.2, 1.3, 1.4, and 1.5, respectively. The surface temperature distribution diagram shown in Figure 10 and the “*y*-$\left|\vartheta \right|$” diagram shown in Figure 11 can be obtained by ANSYS and MATLAB.

According to the results in Figure 11, their temperature change trends are almost the same. When *ka* gradually increases, the peak temperature gradually increases, but the change range is small. In *y*∈(−2, −0) and *y*∈(0, 2), the larger the incident wave number *ka* without diffusion, the greater the change rate of temperature. Under the condition of meeting the temperature requirement, the value of the non-diffusion incident wave number *ka* can be appropriately reduced.

**Figure 12:**
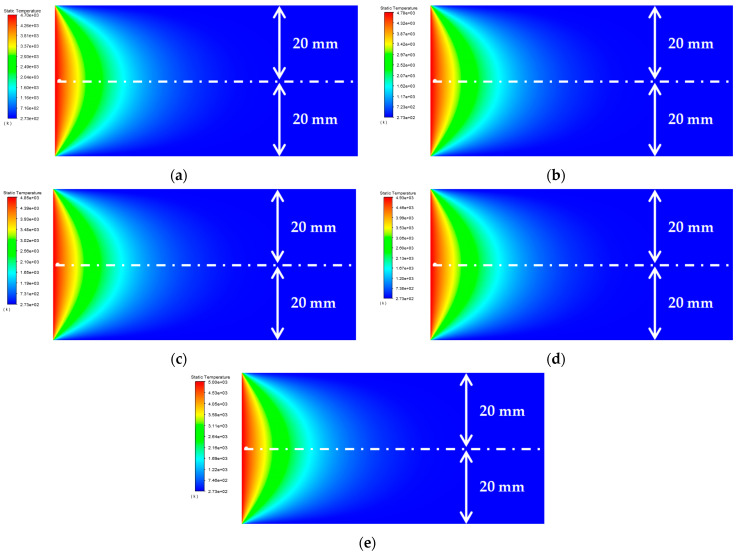
Surface temperature distribution diagram of platinum rhodium bushing: (**a**) *μ*/*a* = 1.1 mm; (**b**) *μ*/*a* = 1.2 mm; (**c**) *μ*/*a* = 1.3 mm; (**d**) *μ*/*a* = 1.4 mm; (**e**) *μ*/*a* = 1.5 mm.

**Figure 13:**
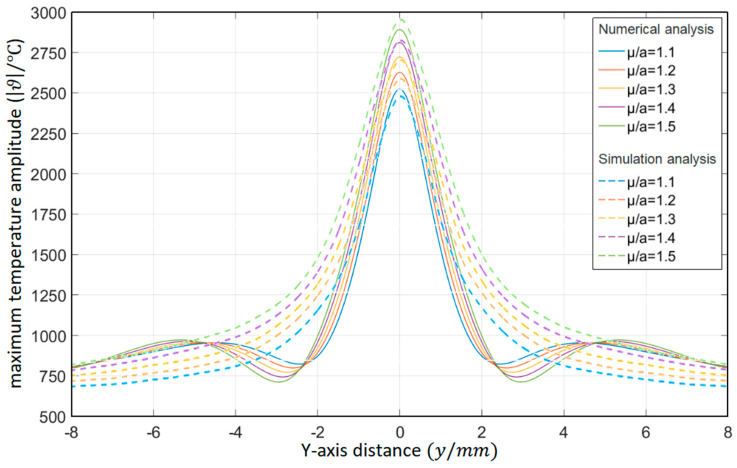
Influence of the relative thermal diffusion length on the temperature distribution in the *Y*-axis direction (ANSYS).

As shown in Table 6, the relative thermal diffusion length is *μ*/*a* = 1.1, 1.2, 1.3, 1.4, and 1.5, respectively. The surface temperature distribution diagram shown in Figure 12 and the “*y*-$\left|\vartheta \right|$” diagram shown in Figure 13 can be obtained by ANSYS and MATLAB.

According to the results in Figure 13, their temperature change trends are almost the same. When *μ*/*a* gradually increases, the peak temperature gradually increases. When the temperature requirement is met, the value of the relative thermal diffusion length *μ*/*a* can be appropriately reduced.

## 8. Conclusions

In this paper, a semi-infinite lath structure model is constructed, and the expression of surface temperature distribution of an Pt–Rh alloy plate with a circular through hole is obtained based on the non-Fourier heat conduction equation, complex function method and conformal mapping method. By controlling the position of the circular through hole and the frequency and amplitude of the laser pulse, the surface temperature distribution of the round through hole in the Pt–Rh bushing at different positions and when the incident light source adopts different non-diffusion incident wave number and relative thermal diffusion length is obtained. The following conclusions can be drawn:

(1) The fracture phenomenon is more likely to occur at the through hole where heat is concentrated.
(2) When the circular through hole is located at the center line of the Pt–Rh bushing, the temperature distribution is symmetrical about the center line of the Pt–Rh bushing, the temperature reaches the peak right in front of the circular through hole, and the peak temperature has nothing to do with the width of the Pt–Rh bushing; however, the temperature change rate becomes slower with the increase of the width of the Pt–Rh bushing. This is because the farther the distance between the circular through hole and the upper and lower surfaces of the Pt–Rh bushing plate, the lower the heat exchange with the external environment, and thus the temperature changes relatively slowly.
(3) When the circular through hole deviates from the center line of the Pt–Rh bushing, the temperature distribution is no longer symmetrical, but the peak temperature is still obtained in front of the circular through hole. When the distance from the circular through hole to the side surface of the Pt–Rh bushing is fixed, the peak temperature decreases with the increase of the width of the Pt–Rh bushing, that is, the increase of the offset of the circular through hole. When opening a hole in the Pt–Rh bushing, the circular through hole can be set at a position deviated from the center line of the Pt–Rh bushing, thus avoiding the excessive superimposed temperature at the circular through hole.
(4) The buried depth of the circular through hole significantly affects the peak temperature. The shallower the buried depth of the circular through hole, the higher the peak temperature. When the buried depth of the circular through hole exceeds a certain range, the temperature distribution on the surface of the Pt–Rh bushing is almost the same as that on the surface of the Pt–Rh bushing without through hole, there is almost no fluctuation in the *Y*-axis direction, and the phenomenon of heat concentration tends to disappear.
(5) When the short wave is incident, the heat propagation characteristic is granular, and with the increase of the wave number, the temperature at the circular through hole also increases. When the incident wave is long, the fluctuation characteristics of heat propagation are weak, and the calculated results of temperature are approximately similar to those based on the thermal diffusion equation. Therefore, the classical heat conduction equation can be used for analysis and calculation.
(6) When the thermal diffusion length is long, the fluctuation characteristics of heat conduction have a great influence on the temperature. In addition, when the thermal diffusion length increases, the peak temperature gradually increases.

Comparing the numerical analysis results with the ANSYS thermal simulation results, it is found that the temperature change trends of both are almost the same. According to the surface temperature distribution expression of the semi-infinite lath structure proposed in this paper, the optimal range of experimental parameters can be determined efficiently before the experiment and simulation, which can effectively reduce the loss of precious metal materials and the time of thermal simulation in the experimental process. This has important reference significance for structural design, quality inspection, process optimization, and service life improvement of Pt–Rh bushings, and also provides a certain reference value for other related research of metal materials with perforated plate structures.

## Figures and Tables

**Table 1:** Related parameters of Pt–Rh alloy with a rhodium content of 10%.

Density (*ρ*)	Thermal Conductivity (*λ*)	Specific Heat Capacity (*c_p_*)	Thermal Relaxation Time (*τ*)
20.5 g/cm^3^	70.05 W/(m·K)	0.133 kJ/(kg·K)	10^−12^ s

**Table 2:** Parameters of circular hole and incident light source in the first simulation.

	Radius of Hole (*a*)	Buried Depth (*b*)	Upper Surface Distance (*c*_1_)	Lower Surface Distance (*c*_2_)	Non-Diffusion Incident Wave Number (*ka*)	Relative Thermal Diffusion Length (*μ/a*)
1	1 mm	1.2 mm	10 mm	10 mm	1.1	1.1
2			15 mm	15 mm		
3			20 mm	20 mm		
4			25 mm	25 mm		
5			30 mm	30 mm		

**Table 3:** Parameters of circular hole and incident light source in the second simulation.

	Radius of Hole (*a*)	Buried Depth (*b*)	Upper Surface Distance (*c*_1_)	Lower Surface Distance (*c*_2_)	Non-Diffusion Incident Wave Number (*ka*)	Relative Thermal Diffusion Length (*μ/a*)
1	1 mm	1.2 mm	20 mm	20 mm	1.1	1.1
2				25 mm		
3				30 mm		
4				35 mm		
5				40 mm		

**Table 4:** Parameters of circular hole and incident light source in the third simulation.

	Radius of Hole (*a*)	Buried Depth (*b*)	Upper Surface Distance (*c*_1_)	Lower Surface Distance (*c*_2_)	Non-Diffusion Incident Wave Number (*ka*)	Relative Thermal Diffusion Length (*μ/a*)
1	1 mm	1.1 mm	20 mm	20 mm	1.1	1.1
2		1.2 mm				
3		1.3 mm				
4		1.4 mm				
5		1.5 mm				

**Table 5:** Parameters of circular hole and incident light source in the fourtht simulation.

	Radius of Hole (*a*)	Buried Depth (*b*)	Upper Surface Distance (*c*_1_)	Lower Surface Distance (*c*_2_)	Non-Diffusion Incident Wave Number (*ka*)	Relative Thermal Diffusion Length (*μ/a*)
1	1 mm	1.2 mm	20 mm	20 mm	1.1	1.1
2					1.2	
3					1.3	
4					1.4	
5					1.5	

**Table 6:** Parameters of circular hole and incident light source in the fifth simulation.

	Radius of Hole (*a*)	Buried Depth (*b*)	Upper Surface Distance (*c*_1_)	Lower Surface Distance (*c*_2_)	Non-Diffusion Incident Wave Number (*ka*)	Relative Thermal Diffusion Length (*μ/a*)
1	1 mm	1.2 mm	20 mm	20 mm	1.1	1.1
2						1.2
3						1.3
4						1.4
5						1.5

## Data Availability

Not applicable.
